# Effect of a Multi-specialty E-consultation Service on Hospital Referrals: An Interrupted Time-Series Study in the Netherlands

**DOI:** 10.1007/s11606-026-10485-5

**Published:** 2026-05-19

**Authors:** Ken M. M. Peeters, Dennis M. J. Muris, Luc G. Gidding, Karin Hek, Mariëlle Krekels, Jochen W. L. Cals

**Affiliations:** 1 Department of Family Medicine, Care and Public Health Research Institute (CAPHRI), Maastricht University, Peter Debyeplein 1, 6229 HA Maastricht, The Netherlands; 2 Zuyderland Medical Centre, Sittard, The Netherlands; 3 Medical Coordination Centre Omnes, Sittard, The Netherlands; 4 GGD Zuid-Limburg (Public Health Institute South-Limburg), Heerlen, The Netherlands; 5 Nivel (Netherlands Institute for Health Services Research), Utrecht, The Netherlands

**Keywords:** e-consultation, e-consult, electronic consultation

## Abstract

**Background:**

E-consultation services between family physicians and hospital specialists may help manage rising healthcare costs and improve care accessibility. Yet, robust evaluations of e-consultation impact on hospital referral patterns remain scarce.

**Objective:**

To evaluate the effect of a multi-specialty e-consultation service between family physicians and medical specialists on hospital referral rates.

**Design:**

An interrupted time series analysis was conducted in the Netherlands between 2015 and 2023, including family practices that submitted e-consultations to 11 specialties at a regional hospital.

**Participants:**

Family practices from a specific region in the Netherlands that used the e-consultation service were included as the exposed group, and a national sample of family practices from the Nivel Primary Care Database served as the control group.

**Intervention:**

Implementation of a multi-specialty e-consultation service.

**Main Measures:**

Monthly referral rates for outpatient clinic visits were analyzed utilizing seasonal autoregressive integrated moving average (SARIMA) models and difference-in-differences analyses to evaluate trends before and after the introduction of the e-consultation service. Sensitivity analyses were performed using various lag periods, and subgroup analyses were conducted based on e-consultation volumes and family physician experience.

**Key Results:**

There was a limited overall impact of e-consultations on hospital referral trends. Internal Medicine showed a regional reduction in referrals (−1.56% per month, *p* < 0.01), though this was not significant compared to national trends (difference-in-difference: 0.68%, *p* = 0.24). Surgery and Neurology showed significant regional increases (Surgery: + 1.76% per month, *p* < 0.01; Neurology: + 1.75% per month, *p* = 0.01), which were also significant compared to national trends in the difference-in-difference analysis (Surgery: −1.73%, *p* < 0.01; Neurology: −1.81%, *p* = 0.01). Subgroup and sensitivity analyses did not reveal consistent patterns or delayed effects.

**Conclusions:**

Specialties did not show a clinically meaningful reduction in referrals, likely due to the relatively small proportion of e-consultations compared to regular referrals. While the overall effect was limited, targeted implementation of e-consultations within specific contexts may help optimize referral practices. Further research is needed to explore the contextual factors that influence the effectiveness of e-consultations.

**Supplementary Information:**

The online version contains supplementary material available at https://doi.org/10.1007/s11606-026-10485-5.

## INTRODUCTION

Healthcare usage and costs are rising in Western countries and are projected to increase even further due to the aging population, earlier detection of diseases, more expensive medical technologies, and longer treatment durations^1^. Healthcare professionals are seeking ways to manage these growing costs while simultaneously maintaining or improving the quality of care. A substantial portion of healthcare expenditures is attributed to hospital care^1,2^. Electronic consultations (e-consultations), a form of asynchronous electronic communication between family physicians (FPs) and medical specialists, have the potential to reduce the number of hospital outpatient referrals^3,4^, as they enable FPs to manage the patient themselves with specialist advice obtained digitally. This reduction can contribute to keeping our healthcare accessible and managing healthcare costs.

Previous studies have shown heterogeneous results^5^. Moreover, there is a lack of (quasi-)experimental research as most existing studies are observational. The majority of this research has been conducted in North America, with only few studies originating from Europe^5^. Healthcare in the USA and Canada is differently organized and serves different populations than smaller, densely populated countries like the Netherlands. Many European countries have universal healthcare systems that ensure access for all, though they are challenged by rising costs due to aging populations. In the Netherlands FPs act as gatekeepers to specialist care and play a central role in the e-consultation process, unlike in North America, where not all patients have a regular FP. Most of the existing literature focuses on the follow-up after e-consultation, specifically whether it can prevent a referral or, conversely, lead to an additional referral due to a potential inductive effect^4,5^. While these individual outcomes are important, they do not capture the broader impact of e-consultations on healthcare utilization. E-consultations are increasingly viewed as a tool to maintain accessibility and affordability of care. Therefore, it is essential to examine their effect on total referral rates over time, rather than solely evaluating the outcome of individual cases. A robust, large-scale, multi-specialty evaluation is lacking. This study aims to evaluate the impact of e-consultation for multiple specialties on hospital referrals. We also aim to explore whether the impact on referral rates is different for specific specialties, or is influenced by monthly e-consultation volumes per FP practice, and FP experience. To our knowledge, our study is the first quasi-experimental study to assess these factors in a multi-specialty setting.

## METHODS

### Design, Participants and Setting

We used a natural pre-post quasi-experimental design to study the number of hospital referrals before and after the implementation of e-consultation. Referral data of family practices to the 11 hospital specialties of the Zuyderland Medical Centre, located in Sittard-Geleen that received the most e-consultations, were collected: Internal Medicine, Gastro-Enterology, Cardiology, Neurology, Pediatrics, Gynecology, Pulmonology, Surgery, Orthopedics and Urology.

### Intervention

E-consultation is a form of asynchronous electronic communication between FPs and medical specialists. FPs ask a specific clinical question or seek advice about the care of a particular patient through a secured digital platform, after which the specialist submits an answer to the FP within five working days^3^. E-consultation is unidirectional and the communication between the FP and specialist is stored in a shared and secured electronic record. In numerous regions of the Netherlands, FPs currently have the ability to submit e-consultations to specialists in participating hospitals. Our research focused on the “Westelijke Mijnstreek and Echt” region, surrounding the Zuyderland Medical Centre. This area, with approximately 190,000 inhabitants in 2023, is a pioneer in the Netherlands regarding e-consultation, as there it was introduced in 2016 by the regional transmural organization Medical Coordination Centre (MCC) Omnes. Consequently, e-consultation has been offered for an extended period, with most FPs utilizing it, and all hospital specialties being accessible for consultation. In the Dutch healthcare system, e-consultations between general practitioners and medical specialists are not directly billed to patients, as they take place within the existing reimbursement structures of insured care.

### Measurements

The primary outcome was defined as the trend of the total number of hospital referrals per 1000 registered patients in family practices per month before and after the introduction of the e-consultation, for the 11 hospital specialties that received the most e-consultations. The total numbers of e-consultations and hospital referrals requested by FPs were calculated for each month and divided by the total number of enlisted patients in the region, multiplied by 1000. This regional normalization accounted for population size and enabled consistent comparisons over time. The moment of implementation of e-consultation varied across specialties, ranging from 2016 to 2020. For each specialty, the implementation date was defined as the date of the first recorded e-consultation in the regional dataset. The research data ranged from 2015 through 2023. The study region included 56 family practices, covering around 190,000 registered patients. To account for expected seasonal variation, such as a higher number of monthly referrals due to seasonal illnesses, the data were aggregated per month to enable statistical control. This approach ensured that monthly fluctuations were not incorrectly attributed to the introduction of the e-consultation.

### Primary Analysis

The primary analysis involved comparing hospital referrals before and after the introduction of e-consultation for each specialty. We calculated slope changes, which represent the monthly change in referrals per 1000 patients; these were expressed as the monthly percentage change relative to the first month of either the pre- or post-intervention period. The control period was the time prior to the implementation of e-consultation. Recognizing that other external factors, such as broader changes in the healthcare system, could also have influenced referral patterns after the intervention, we also incorporated a reference group using national hospital referral data. Since the studied region was an early adopter of e-consultation, other regions in the Netherlands had little to no exposure to this intervention during the analysis period. National referral data from 2015 to 2023 were obtained from family practices participating in the Nivel Primary Care Database^6^, which contains routinely recorded data from a sample of practices across the Netherlands, including over 400 general practices and approximately 1.9 million registered patients. The Nivel Primary Care Database has been shown to be broadly representative of the Dutch population; further details on data collection and representativeness are described elsewhere ^6^. All analyses, including the difference-in-differences approach, were conducted separately for each specialty rather than for overall referral rates across specialties combined. This study has been approved according to the governance code of Nivel Primary Care Database, under number NZR-00325.001.

### Subgroup and Sensitivity Analyses

Subgroup analyses were conducted based on the number of requested e-consultations per month and years of experience of the referring FP. This was done to investigate whether effects differed between FPs who frequently or infrequently requested e-consultations, and between more and less experienced FPs. Subgroups were categorized into tertiles. Additionally, we considered the possibility of a lag period in the effect of e-consultations on referral rates, i.e., the impact of introducing e-consultations might not appear immediately but could emerge after some delay. To account for this, we performed a sensitivity analysis within the regional group, applying lag periods of 12 and 24 months. As a sensitivity analysis for multiple testing, false discovery rate (Benjamini–Hochberg) correction was applied to *p*-values across specialties within each set of analyses (regional slope change, national slope change, and difference-in-difference estimates).

### Statistical Analysis

To assess long-term effects of e-consultation implementation, we employed an interrupted time series (ITS) analysis. ITS is a robust method when randomization is not feasible, allowing for the examination of causal effects in an observational natural experiment. This approach helps identify changes in outcomes by accounting for existing trends in the data, thus minimizing the influence of external factors, assuming these factors remain stable. Therefore, the primary control period for evaluating the referral rate was the trend observed prior to the introduction of e-consultation. To further control for factors that may have changed after the intervention, a reference group was established from regions where e-consultation was not yet implemented or used minimally.

We utilized a seasonal autoregressive integrated moving average (SARIMA) model^7,8^. We compared the trends in consultation rates before and after the implementation of e-consultation by calculating changes in the slope. For each specialty, a single SARIMA model was fitted that included both the regional (intervention) and national (reference) groups, allowing for a difference-in-differences (DiD) analysis. This approach estimates the change in referral trend over time in the intervention group relative to the change observed in the reference group, thereby accounting for shared temporal patterns and external influences. Since SARIMA models assume linearity, we checked for stationarity of the mean and variance over time using differencing. Autocorrelation and seasonality were assessed by inspecting the autocorrelation (ACF) and partial autocorrelation functions (PACF), and appropriate models were selected for each group. After model application, the ACF and PACF of the residuals were examined and tested using the Box-Ljung statistic, confirming that there were no significant autocorrelations in the residuals, which validated the models’ appropriateness. All statistical analyses were conducted using R Statistical Software (version 4.4.3; R Core Team 2025).

## RESULTS

### Referral Rates Before and After Implementation of E-consultation

The implementation of the e-consultation service showed a limited overall impact on hospital referral rates. Table 1 shows that the mean number of e-consultations per 1000 patients per month ranged from 0.25 (± 0.18) for the specialty that received the most e-consultations (Internal Medicine) to 0.02 (± 0.02) for the specialty that received the least (Surgery and Rheumatology). Trends in the referral rates for each specialty before and after the introduction of e-consultation are depicted in Table 2. The model characteristics for each fitted model, including the time period of data used, are shown in Appendix 1.

**Table 1:** Mean Number of e-Consultations and Referrals per 1000 Patients per Month for Each Specialty in the Intervention Region

**Specialty**	**E-consultations** Mean per 1000 patients per month (SD)	**Referrals** Mean per 1000 patients per month (SD)	**Ratio of e-consultations to referrals**
Cardiology	0.05 (0.05)	1.34 (0.71)	0.038
Gastro-enterology	0.07 (0.07)	0.47 (0.26)	0.140
Gynecology	0.04 (0.05)	1.18 (0.63)	0.035
Internal Medicine	0.25 (0.18)	1.13 (0.27)	0.225
Neurology	0.04 (0.05)	1.48 (0.84)	0.030
Orthopedics	0.04 (0.07)	1.91 (1.08)	0.023
Pediatrics	0.03 (0.04)	0.51 (0.29)	0.060
Pulmonology	0.02 (0.03)	0.62 (0.35)	0.040
Rheumatology	0.02 (0.02)	0.45 (0.24)	0.043
Surgery	0.02 (0.02)	1.35 (0.74)	0.013
Urology	0.03 (0.04)	0.99 (0.52)	0.029

**Table 2:** Difference-in-Difference Analysis, Difference in Slope Change in Referral Rates Before and After the Introduction of the e-Consultation Between the “Westelijke Mijnstreek and Echt” Region and the National Reference Group. All Percentage Changes Reflect Monthly Trends Relative to the Initial Referral Rate Within the Respective Period. Referral Rates Are Expressed per 1000 Patients

Specialty	**Regional**			**National**			**Difference-in-difference**
	**Pre-intervention slope** (% per month)	**Post-intervention slope** (% per month)	Slope change* (% per month)	**Pre-intervention slope** (% per month)	**Post-intervention slope** (% per month)	Slope change* (% per month)	Difference in slope change* (%; national – regional)
Cardiology	0.00	−0.15	−0.15 (*p* = 0.74)	0.238	0.122	−0.12 (*p* = 0.86)	0.03 (*p* = 0.74)
Gastro-enterology	1.48	−0.17	−1.66 (*p* = 0.47)	0.530	0.073	−0.46 (*p* = 0.39)	1.20 (*p* = 0.47)
Gynecology	−0.29	−0.07	0.22 (*p* = 0.35)	0.365	0.086	−0.28 (*p* = 0.53)	−0.50 (*p* = 0.35)
Internal Medicine	0.97	−0.59	−1.56* (*p* < 0.01)	0.766	−0.118	−0.88 (*p* = 0.17)	0.68 (*p* = 0.24)
Neurology	−0.90	0.85	1.75* (*p* = 0.01)	0.137	0.078	−0.06 (*p* = 0.92)	−1.81* (*p* = 0.01)
Orthopedics	−0.64	−0.27	0.36 (*p* = 0.59)	−0.332	0.428	0.76 (*p* = 0.09)	0.40 (*p* = 0.59)
Pediatrics	−0.34	−0.29	0.05 (*p* = 0.92)	0.092	0.117	0.03 (*p* = 0.95)	−0.02 (*p* = 0.92)
Pulmonology	−0.19	−0.18	0.01 (*p* = 0.66)	0.046	0.385	0.34 (*p* = 0.60)	0.33 (*p* = 0.66)
Rheumatology	0.08	−0.01	−0.09 (*p* = 0.16)	0.178	0.330	0.15 (*p* = 0.20)	0.24 (*p* = 0.16)
Surgery	−0.85	0.91	1.76* (*p* < 0.01)	0.297	0.324	0.03 (*p* = 0.88)	−1.73* (*p* < 0.01)
Urology	−0.03	0.04	0.07 (*p* = 0.42)	−0.062	0.420	0.48 (*p* = 0.17)	0.41 (*p* = 0.42)

^*^Significant if *p* < 0.05

**Figure 1:**
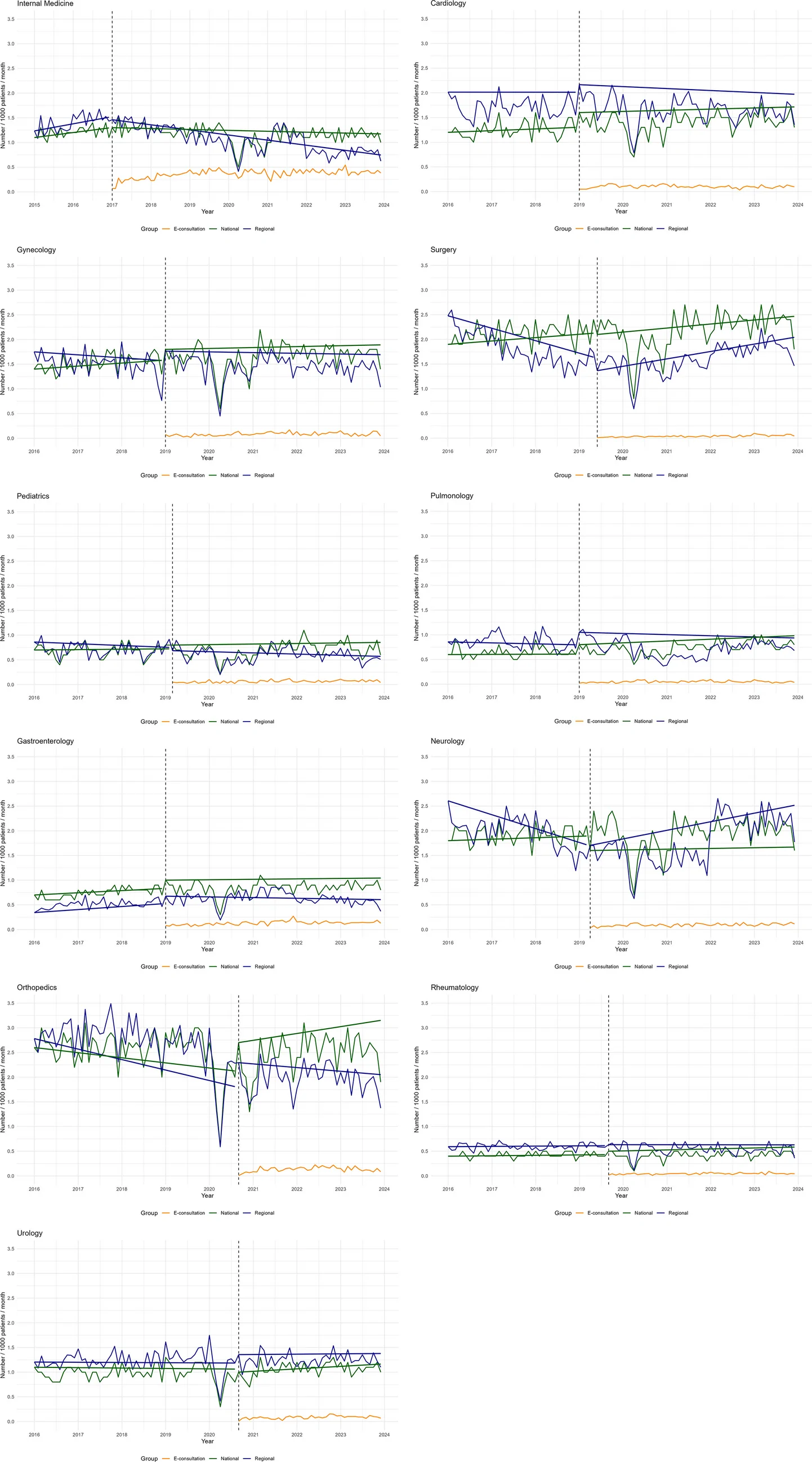
Change in referral rates before and after the introduction of the e-consultation (orange line) for the “Westelijke Mijnstreek and Echt” region (blue line) and the national reference group (green line).

Regionally, Internal Medicine showed a significant decrease (−1.56% per month, *p* < 0.01) after introduction of the e-consultation (Fig. 1). The mean number of referrals for this specialty was 1.13 referrals per 1000 patients per month (± 0.27). The national group showed no significant trend change for this specialty (−0.88% per month, *p* = 0.17). The difference in trend change between the regional group and the national group was not significant (0.68% greater decrease per month in the regional group compared to the national group, *p* = 0.24). The specialties of Surgery and Neurology showed significant increases in referral rates in the intervention region (Surgery: 1.76%, *p* < 0.01; Neurology: 1.75%, *p* = 0.01), contrary to non-significant national trends (Surgery: 0.03%, *p* = 0.88; Neurology: −0.06%, *p* = 0.92). The difference-in-differences analysis revealed significant differences in slope changes for both specialties (Surgery: −1.73%, *p* < 0.01; Neurology: −1.81%, *p* = 0.01).

### Sensitivity and Subgroup Analyses

The sensitivity analysis showed that the direction and magnitude of the intervention effect were similar for both lag periods and largely consistent with the primary analysis. Subgroup analyses based on practice volume and FP experience did not reveal any consistent patterns across specialties. After false discovery rate correction, none of the previously observed statistically significant effects remained significant (Appendix 2).

## DISCUSSION AND CONCLUSION

This study evaluated the impact of a multi-specialty e-consultation service on hospital referral rates within the Netherlands using an interrupted time series analysis. Overall, the introduction of e-consultation showed minimal impact on trends in hospital referral rates across the studied specialties. Significant differences were observed in specific specialties. Internal Medicine, with the highest volume of e-consultations, demonstrated a significant reduction in regional referrals, though this did not remain significant when compared to national trends. Conversely, Surgery and Neurology specialties showed unexpected but non-significant increases in regional referrals, contrary to non-significant national trends. These differences were statistically significant in the difference-in-difference analysis.

These findings expand upon existing knowledge of e-consultation impact on hospital referrals. Two Spanish studies applied an interrupted time series design to evaluate e-consultations for Cardiology. Both used regression-based models rather than SARIMA and focused on hospital admissions. The first study reported improved accessibility but also an increase in care demand^9^, whereas the second found a significant reduction in hospital admissions^10^. Several other studies have used pre-post designs, typically focusing on the number of e-consultations and their immediate follow-up, without employing ITS methods^5^. Our study uniquely contributes to the body of evidence by employing robust SARIMA modeling to comprehensively evaluate longitudinal referral trends across multiple medical specialties, explicitly accounting for autocorrelation and seasonality in time series data.

For Internal Medicine, a significant difference was observed in the regional model, although this difference was no longer statistically significant in the difference-in-differences analysis compared to the national control group. Notably, Internal Medicine also had the highest number of e-consultations. The regional reduction in the slope for Internal Medicine was −1.56% referrals per 1000 patients per month. This decrease is substantial and could therefore be considered clinically meaningful. To illustrate the potential magnitude of this effect, based on an average referral rate of approximately 1.13 referrals per 1000 patients per month and a regional population of around 190,000 inhabitants, this trend could correspond to an estimated reduction of approximately 30–40 referrals per year.

Other specialties did not show clinically meaningful differences, likely because the proportion of e-consultations relative to regular referrals was too small. In fact, for several specialties, the proportion of e-consultations relative to referrals was so small that any meaningful effect on referral trends would have been implausible from the outset. Previous research in the Netherlands has shown a high number of avoided referrals for Internal Medicine^11^. These findings may be partly explained by the diagnostic nature of Internal Medicine^3^. Uncertainty around diagnoses often prompts FPs to seek specialist input. Moreover, this specialty frequently manages chronic or general conditions that are suitable for remote care, such as through medication or lifestyle adjustments. A more conservative approach, focusing on monitoring rather than immediate referral, may also contribute. International literature does not demonstrate a higher number of avoided referrals following e-consultation for Internal Medicine compared to other specialties^4,5^. One possible explanation is that not only the inherent characteristics of the specialty but also the implementation context plays a crucial role. Factors such as the degree of engagement of medical specialists, the quality and level of detail of specialist answers, and the pioneering role of specific specialties may all influence the observed outcomes. Therefore, further implementation research is required to explore these contextual influences, ideally over longer periods of time.

Interestingly, there was a significant increase in referral rates for Surgery and Neurology in the “Westelijke Mijnstreek and Echt” region, which was not observed in the national control group. It is very unlikely that e-consultations themselves have led to an increase in referrals, given previous research regarding the potential inductive effect of e-consultation^5,12^. Studies have shown that e-consultations help to avoid substantially more referrals than they generate. Moreover, the absolute number of e-consultations addressed to these specialties in our study was relatively low, further reducing the likelihood that they account for the observed increase in referral rates. This suggests that additional factors may influence these trends. As previous qualitative research has shown, factors may include regional differences in patient case-mix, access to specialist care and waiting times, hospital-specific policies, physicians’ referral behavior, the quality of interprofessional collaboration, and local workflow or implementation arrangements. In the context of our interrupted time series design, such factors are particularly relevant if they changed differentially over time in one group.

Our sensitivity analyses using alternative lag periods demonstrated that, for most specialties, the intervention effects remained consistent, reinforcing the robustness of our primary findings. There is no evidence of a delayed or ceiling effect. Although subgroup analysis was performed, no consistent differences were found between more and less experienced FPs, indicating that experience level and e-consultation volume may not substantially influence the effect of e-consultation on referral behavior.

### Implications

While the overall effects on referral rates were limited, the observed reduction in referrals for Internal Medicine may be clinically meaningful, although the lack of statistical significance in the national comparison suggests that this finding should be interpreted with caution. For most other specialties, the number of e-consultations was relatively low, which may have limited the ability to detect significant effects. If the volume of e-consultations increases over time, similar effects might emerge in other specialties. It is worth noting that Internal Medicine was the first specialty to implement e-consultations, suggesting that maturity of implementation could influence effectiveness. Several contextual factors may impact the uptake and effectiveness of e-consultations, including specialist engagement, the quality and level of detail in responses, and the notoriety of the service. Targeted strategies, such as improving notoriety, enhancing engagement, and developing clear working agreements on how to structure e-consult questions and answers, may help optimize usage. Furthermore, targeted training sessions (e.g., continuing education on how to effectively use e-consultations) could enhance impact. The primary value of e-consultation may not lie in cost reduction. Previous qualitative studies have emphasized its broader benefits, such as improved access to specialist advice, support for FPs, enhanced communication, and better documentation^3^. Nonetheless, potential downsides should also be considered, such as the risk of over-reliance on remote advice leading to missed diagnoses, or a possible inductive effect in which specialist input prompts additional referrals that might not otherwise have been made. However, previous studies have indicated that these effects are minimal^4^, and it should be acknowledged that referrals are often necessary and appropriate from the perspective of providing high-quality patient care. Reducing referral rates is therefore not inherently desirable in all cases.

### Strengths and Limitations

A major strength of this study is the use of a pre-post design combined with a control group. Analyses are based on a large dataset, covering multiple specialties, and spanning several years, which enhances the robustness and generalizability of findings. This design was the most feasible option, as conducting a randomized controlled trial (RCT) was not possible or practical. It would not have been desirable to withhold access to e-consultation for certain FP practices, especially since it was rapidly implemented across many specialties during the COVID-19 pandemic, when the need for such services became particularly critical. Interrupted time series designs are widely regarded as one of the strongest quasi-experimental approaches for evaluating population-level healthcare interventions when randomized controlled trials are infeasible, particularly when accounting for underlying trends and autocorrelation^13–18^.

However, one limitation is the lack of precise information regarding the implementation and volume of e-consultations in other regions. Although a control group was included, fully accounting for the influence of other confounding factors remains challenging. Previous qualitative research has shown that contextual factors may influence whether e-consultations translate into changes in referral behavior^3,19–22^. The ITS approach assumes that these factors remain relatively stable within each group or change similarly over time in both the intervention and reference groups. If such factors changed differentially between the intervention and reference regions during the study period, this could potentially influence referral patterns and therefore affect the estimated intervention effect.

Furthermore, it is unclear if the family practices participating in the Nivel Primary Care database made any use of e-consultations. Nevertheless, as our analyses spanned multiple years and the uptake of e-consultations in other regions appears to have occurred only in the most recent period and in small number, any potential impact on the findings is likely to be limited.

Another important limitation is the impact of the COVID-19 pandemic, which may have affected referral rates. During this period, referral volumes decreased across specialties in both the regional and national reference groups, while no marked increase in e-consultations was observed. This suggests that pandemic-related disruptions likely influenced both groups in a similar way, although regional differences in how care was organized during this period cannot be fully excluded. Our primary analyses were conducted separately for each medical specialty, based on the assumption that referral patterns, e-consultation implementation, and clinical context vary meaningfully between specialties. Consequently, we did not apply correction across specialties, as this could increase the risk of type II errors and obscure clinically relevant effects in specific domains. We acknowledge, however, that multiple testing increases the risk of type I errors, and our findings should be interpreted with appropriate caution, especially for marginal *p*-values^23^. As a sensitivity analysis, false discovery rate–adjusted *p*-values are presented in Appendix 2. Subgroup and sensitivity analyses yielded no statistically significant results at *α* = 0.05, meaning that correction for multiple comparisons would not have altered the interpretation of these findings.

The generalizability of these findings to other countries depends largely on the structure of the healthcare system, particularly the role of the FP as a gatekeeper to specialist care. In systems where FPs play a central role in managing referrals, such as in the Netherlands, e-consultations may influence referral rates differently than in systems with more direct access to specialists. In countries where access to specialist care is limited, whether due to geographic barriers (e.g., sparsely populated areas with long travel distances) or financial constraints, e-consultations may be used for different types of clinical questions and may have a different impact on referral behavior.

Our primary outcome, total referral rates, may not fully capture the impact of e-consultations on referral appropriateness. If e-consultations mainly prevent unnecessary referrals, effects may be diluted when analyzing overall referral rates, particularly given the relatively low uptake in our study. As we could not distinguish between appropriate and inappropriate referrals using routine data, future studies should incorporate measures of referral appropriateness (e.g., chart review or proxy indicators) to better assess their impact on low-value care.

Future studies with larger datasets and higher e-consultation volumes may allow more detailed analyses at the level of individual physicians or patient subgroups to better understand for which contexts e-consultation is most effective. Furthermore, a formal economic evaluation was beyond the scope of this study, but future research should examine the cost-effectiveness of e-consultation services, including potential effects on healthcare expenditures, patient travel costs, and physician workload.

## CONCLUSION

The implementation of a multi-specialty e-consultation service exhibited limited overall influence on hospital referral trends. Although isolated specialties showed significant changes, notably Internal Medicine with reduced referrals, the service did not systematically alter referral patterns across specialties, likely due to low e-consultation volumes in most specialties. Targeted implementation in specific contexts may optimize referral practices. Further research into the contextual factors influencing e-consultation effectiveness is needed.

## Supplementary Information

Below is the link to the electronic supplementary material.

Supplementary file1 (DOCX 20.8 KB)

## Data Availability

Data are available from the corresponding author upon reasonable request, subject to applicable privacy and ethical restrictions.

## Abbreviations

FP: Family physician

## References

[1] Commision OE. Health at a Glance: Europe 2024: State of Health in the EU Cycle. OECD Publishing, Paris. 2024.

[2] **Vonk RAA HH, Plasmans MHD, Kommer GJ, Polder JJ.** Health care expenditures foresight 2015–2060 : Quantitative preliminary study at the request of the Scientific Council for Government Policy (WRR). Part 1: future projections Bilthoven, the Netherlands: Rijksinstituut voor Volksgezondheid en Milieu RIVM; 2020.

[3] **Peeters K, Giroldi E, Veldhuizen L, Abdelaziz K, Muris D, Cals J.** General Practitioner Use of e-Consultation to Consult Hospital Specialists: Interview Study to Obtain Physician's Perceptions About Digital Interprofessional Communication. J Med Internet Res. 2023;25:e40318. 10.2196/40318 PMC10131626 36976626

[4] **Vimalananda VG, Orlander JD, Afable MK, Fincke BG, Solch AK, Rinne ST, et al.** Electronic consultations (E-consults) and their outcomes: a systematic review. J Am Med Inform Assoc. 2020;27(3):471-9. 10.1093/jamia/ocz185 PMC7647247 31621847

[5] **Peeters KMM, Reichel LAM, Muris DMJ, Cals JWL.** Family Physician-to-Hospital Specialist Electronic Consultation and Access to Hospital Care: A Systematic Review. JAMA Netw Open. 2024;7(1):e2351623. 10.1001/jamanetworkopen.2023.51623 PMC10787322 38214930

[6] **Vanhommerig JW, Verheij RA, Hek K, Ramerman L, Hooiveld M, Veldhuijzen NJ, et al.** Data Resource Profile: Nivel Primary Care Database (Nivel-PCD), The Netherlands. Int J Epidemiol. 2025;54(2). 10.1093/ije/dyaf017 PMC11906400 40083190

[7] **Lopez Bernal J, Cummins S, Gasparrini A.** The use of controls in interrupted time series studies of public health interventions. Int J Epidemiol. 2018;47(6):2082-93. 10.1093/ije/dyy135 29982445

[8] **Jandoc R, Burden AM, Mamdani M, Lévesque LE, Cadarette SM.** Interrupted time series analysis in drug utilization research is increasing: systematic review and recommendations. J Clin Epidemiol. 2015;68(8):950-6. 10.1016/j.jclinepi.2014.12.018 25890805

[9] **Mazón-Ramos P, Cinza-Sanjurjo S, Garcia-Vega D, Portela-Romero M, Sanmartin-Pena JC, Rey-Aldana D, et al.** A clinician-to-clinician universal electronic consultation programme at the cardiology department of a Galician healthcare area improves healthcare accessibility and outcomes in elderly patients. Eur Heart J Digit Health. 2023;4(2):90-8. 10.1093/ehjdh/ztad004 PMC10039426 36974264

[10] **Cinza-Sanjurjo S, Mazón-Ramos P, Rey-Aldana D, Garcia-Vega D, Portela-Romero M, Rodríguez-Mañero M, et al.** Enhancing patient outcomes: Integrating electronic cardiology consultation in primary care for cancer patients. Eur J Clin Invest. 2024;54(8):e14197. 10.1111/eci.14197 38519859

[11] **Muris D, Krekels M, Spreeuwenberg A, Blom M, Bergmans P, Cals JWL.** [General practitioners’ use of internal medicine e-consultations]. Ned Tijdschr Geneeskd. 2020;164. 32186815

[12] **Liddy C, Drosinis P, Keely E.** Electronic consultation systems: worldwide prevalence and their impact on patient care-a systematic review. Fam Pract. 2016;33(3):274-85. 10.1093/fampra/cmw024 27075028

[13] **Bernal JL, Cummins S, Gasparrini A.** Interrupted time series regression for the evaluation of public health interventions: a tutorial. International Journal of Epidemiology. 2016;46(1):348-55. 10.1093/ije/dyw098 PMC5407170 27283160

[14] **Ewusie JE, Soobiah C, Blondal E, Beyene J, Thabane L, Hamid JS.** Methods, Applications and Challenges in the Analysis of Interrupted Time Series Data: A Scoping Review. Journal of Multidisciplinary Healthcare. 2020;13(null):411–23. 10.2147/JMDH.S241085 PMC7231782 32494150

[15] **Hategeka C, Ruton H, Karamouzian M, Lynd LD, Law MR.** Use of interrupted time series methods in the evaluation of health system quality improvement interventions: a methodological systematic review. BMJ Glob Health. 2020;5(10). 10.1136/bmjgh-2020-003567 PMC7559052 33055094

[16] **Jiang H, Rehm J, Tran A, Lange S.** Interrupted Time Series Design and Analyses in Health Policy Assessment. medRxiv. 2024.

[17] **Korevaar E, Karahalios A, Turner SL, Forbes AB, Taljaard M, Cheng AC, et al.** Methodological systematic review recommends improvements to conduct and reporting when meta-analyzing interrupted time series studies. Journal of Clinical Epidemiology. 2022;145:55-69. 10.1016/j.jclinepi.2022.01.010 35045318

[18] **Schaffer AL, Dobbins TA, Pearson S-A.** Interrupted time series analysis using autoregressive integrated moving average (ARIMA) models: a guide for evaluating large-scale health interventions. BMC Medical Research Methodology. 2021;21(1):58. 10.1186/s12874-021-01235-8 PMC7986567 33752604

[19] **Bello AK, Molzahn AE, Girard LP, Osman MA, Okpechi IG, Glassford J, et al.** Patient and provider perspectives on the design and implementation of an electronic consultation system for kidney care delivery in Canada: a focus group study. BMJ Open. 2017;7(3):e014784. 10.1136/bmjopen-2016-014784 PMC5353303 28255097

[20] **Keely E, Drosinis P, Afkham A, Liddy C.** Perspectives of Champlain BASE Specialist Physicians: Their Motivation, Experiences and Recommendations for Providing eConsultations to Primary Care Providers. Stud Health Technol Inform. 2015;209:38-45. 25980703

[21] **Liddy C, de Man G, Moroz I, Afkham A, Mercer J, Keely E.** Effective Integration of an eConsult Service into an Existing Referral Workflow Within a Primary Care Clinic. Telemed J E Health. 2020;26(5):659-64. 10.1089/tmj.2019.0014 31414979

[22] **Liddy C, Moroz I, Mihan A, Keely E.** Assessment of the Generalizability of an eConsult Service through Implementation in a New Health Region. J Am Board Fam Med. 2019;32(2):146-57. 10.3122/jabfm.2019.02.180169 30850451

[23] **Groenwold RHH, Goeman JJ, Cessie SL, Dekkers OM.** Multiple testing: when is many too much? Eur J Endocrinol. 2021;184(3):E11-e4. 10.1530/EJE-20-1375 33300887

