# Supplementary material for: Effect of a Multi-specialty E-consultation Service on Hospital Referrals: An Interrupted Time-Series Study in the Netherlands
Source: J Gen Intern Med. 2026 May 19;41(13):3749–56. doi: 10.1007/s11606-026-10485-5 (PMC13631177; doi:10.1007/s11606-026-10485-5)
Supplement: Supplementary file 1 [file 11606_2026_10485_MOESM1_ESM.docx]

**Appendix 1. Model characteristics.**

| **Department** | **Time span** | **Model parameters** | **AIC** |
| --- | --- | --- | --- |
| **Cardiology** | Pre: jan 2016 - dec 2018  Post: jan 2019 - dec 2023 | SARIMA(0,0,2) x (2,0,0)[12] | -75 |
| **Gastro-enterology** | Pre: jan 2016 - dec 2018  Post: jan 2019 - dec 2023 | SARIMA(2,0,0) x (1,0,0)[12] | -305 |
| **Gynecology** | Pre: jan 2016 - dec 2018  Post: jan 2019 - dec 2023 | SARIMA(1,0,0) x (2,0,0)[12] | -51 |
| **Internal Medicine** | Pre: jan 2015 - dec 2016  Post: jan 2017 - dec 2023 | SARIMA(2,0,0) x (2,0,0)[12] | -206 |
| **Neurology** | Pre: jan 2016 - mrt 2019  Post: apr 2019 - dec 2023 | SARIMA(2,0,0) x (2,0,0)[12] | 30 |
| **Orthopedics** | Pre: jan 2016 - aug 2020  Post: sep 2020 - dec 2023 | SARIMA(1,0,0) x (2,0,0)[12] | 130 |
| **Pediatrics** | Pre: jan 2016 - feb 2019  Post: mrt 2019 - dec 2023 | SARIMA(2,0,0) x (1,0,0)[12] | -258 |
| **Pulmonology** | Pre: jan 2016 - dec 2018  Post: jan 2019 - dec 2023 | SARIMA(2,0,0) x (2,0,0)[12] | -290 |
| **Rheumatology** | Pre: jan 2016 - aug 2019  Post: sep 2019 - dec 2023 | SARIMA(0,0,3) x (0,0,0)[12] | -424 |
| **Surgery** | Pre: jan 2016 - mei 2019  Post: jun 2019 - dec 2023 | SARIMA(2,0,0) x (2,0,0)[12] | 2 |
| **Urology** | Pre: jan 2016 - aug 2020  Post: sep 2020 - dec 2023 | SARIMA(1,0,0) x (2,0,0)[12] | -163 |

AIC: Akaike Information Criteria. A measure of the quality of the model, which takes both the fit and complexity into account. A lower AIC indicates a better model.

**Appendix 2. Difference-in-differences analysis of changes in referral trends before and after introduction of e-consultation. All reported p-values are false discovery rate (Benjamini–Hochberg) adjusted within each set of comparisons (regional slope change, national slope change, and difference-in-difference estimates). Referral rates are expressed per 1,000 patients.**

|  | **Regional** | | | | **National** | | | | **Difference-in-difference** | |
| --- | --- | --- | --- | --- | --- | --- | --- | --- | --- | --- |
| **Specialty** | **Pre-intervention slope**  (% per month) | **Post-intervention slope**  (% per month) | **Slope change***  (% per month) | **Pre-intervention slope**  (% per month) | | **Post-intervention slope**  (% per month) | **Slope change***  (% per month) | **Difference in slope change***  (%; national – regional) | |  |
| **Cardiology** | 0,00 | -0,15 | -0.15 (p = 0.81) | 0,238 | | 0,122 | -0.12 (p = 0.95) | 0.03 (p = 0.81) | |  |
| **Gastro-enterology** | 1,48 | -0,17 | -1.66 (p = 0.74) | 0,530 | | 0,073 | -0.46 (p = 0.97) | 1.20 (p = 0.77) | |  |
| **Gynecology** | -0,29 | -0,07 | 0.22 (p = 0.64) | 0,365 | | 0,086 | -0.28 (p = 0.97) | -0.50 (p = 0.77) | |  |
| **Internal Medicine** | 0,97 | -0,59 | -1.56 (p = 0.06) | 0,766 | | -0,118 | -0.88 (p = 0.62) | 0.68 (p = 0.66) | |  |
| **Neurology** | -0,90 | 0,85 | 1.75 (p = 0.06) | 0,137 | | 0,078 | -0.06 (p = 0.95) | -1.81 (p = 0.06) | |  |
| **Orthopedics** | -0,64 | -0,27 | 0.36 (p = 0.74) | -0,332 | | 0,428 | 0.76 (p = 0.50) | 0.40 (p = 0.81) | |  |
| **Pediatrics** | -0,34 | -0,29 | 0.05 (p = 0.92) | 0,092 | | 0,117 | 0.03 (p = 0.97) | -0.02 (p = 0.92) | |  |
| **Pulmono-logy** | -0,19 | -0,18 | 0.01 (p = 0.81) | 0,046 | | 0,385 | 0.34 (p = 0.97) | 0.33 (p = 0.81) | |  |
| **Rheumato-logy** | 0,08 | -0,01 | -0.09 (p = 0.44) | 0,178 | | 0,330 | 0.15 (p = 0.62) | 0.24 (p = 0.59) | |  |
| **Surgery** | -0,85 | 0,91 | 1.76* (p = 0.06) | 0,297 | | 0,324 | 0.03 (p = 0.97) | -1.73 (p = 0.06) | |  |
| **Urology** | -0,03 | 0,04 | 0.07 (p = 0.74) | -0,062 | | 0,420 | 0.48 (p = 0.62) | 0.41 (p = 0.77) | |  |

* All p-values are false discovery rate (Benjamini–Hochberg) adjusted within each set of comparisons (regional, national, and difference-in-difference). No comparisons remained statistically significant at α = 0.05 after adjustment.
